# Soluble Receptor for Advanced Glycation End Products and Its Forms in COVID-19 Patients with and without Diabetes Mellitus: A Pilot Study on Their Role as Disease Biomarkers

**DOI:** 10.3390/jcm9113785

**Published:** 2020-11-23

**Authors:** Elena Dozio, Clementina Sitzia, Lara Pistelli, Rosanna Cardani, Roberta Rigolini, Marco Ranucci, Massimiliano M. Corsi Romanelli

**Affiliations:** 1 Department of Biomedical Science for Health, Università degli Studi di Milano, 20133 Milan, Italy; clementina.sitzia@unimi.it (C.S.); mmcorsi@unimi.it (M.M.C.R.); 2 Service of Laboratory Medicine1-Clinical Pathology, IRCCS Policlinico San Donato, San Donato Milanese, 20097 Milan, Italy; lara.pistelli@unimi.it (L.P.); roberta.rigolini@grupposandonato.it (R.R.); 3 Biobank BioCor, Service of Laboratory Medicine1-Clinical Pathology, IRCCS Policlinico San Donato, San Donato Milanese, 20097 Milan, Italy; rosanna.cardani@grupposandonato.it; 4 Department of Cardiothoracic and Vascular Anesthesia and Intensive Care Unit, IRCCS Policlinico San Donato, San Donato Milanese, 20097 Milan, Italy; marco.ranucci@grupposandonato.it

**Keywords:** COVID-19, cRAGE, diabetes mellitus, esRGAE, sRAGE

## Abstract

The receptor for advanced glycation end products (RAGE), a well-known player of diabetes mellitus (DM)-related morbidities, was supposed to be involved in coronavirus disease-19 (COVID-19), but no data exist about COVID-19, DM, and the soluble RAGE (sRAGE) forms. We quantified total sRAGE and its forms, the endogenously secretory esRAGE and the membrane-cleaved cRAGE, in COVID-19 patients with and without DM and in healthy individuals to explore how COVID-19 may affect these molecules and their potential role as biomarkers. Circulating sRAGE and esRAGE were quantified by enzyme-linked-immunosorbent assays. cRAGE was obtained by subtracting esRAGE from total sRAGE. sRAGE, esRAGE, cRAGE, and the cRAGE/esRAGE ratio did not differ between DM and non-DM patients and had the same trend when compared to healthy individuals. Levels of total sRAGE, cRAGE, and cRAGE/esRAGE ratio were upregulated, while esRAGE was downregulated. The lack of difference between DM and non-DM COVID-19 patients in the levels of sRAGE and its forms supports the hypothesis that in COVID-19 the RAGE system is modulated regardless of glycemic control. Identifying how sRAGE and its forms associate to COVID-19 prognosis and the potential of RAGE as a therapeutic target to control inflammatory burden seem of relevance to help treatment of COVID-19.

## 1. Introduction

Receptor for advanced glycation end products (receptor for AGE, RAGE) and its soluble form, sRAGE, are involved in diabetes mellitus (DM)-related diseases [1,2]. RAGE is a membrane-bound receptor expressed at low levels in most human tissues, but with the lungs being the richest ones [3]. The corresponding soluble form, sRAGE, can play a role as a decoy receptor and disease biomarker [4,5,6]. sRAGE is composed of the endogenously secretory form (esRAGE) and the membrane-cleaved form (cRAGE). The first is considered the real decoy receptor, the second a surrogate marker of inflammation [1,2], but both can block ligand from binding to membrane RAGE. Engagement of RAGE increases its expression, activates inflammation, downregulates esRAGE, and promotes RAGE cleavage into cRAGE. In patients with DM, sRAGE has been shown to increase as a counter-regulatory mechanism against AGE, which reaches very high levels due to hyperglycemia [1,2].

RAGE and its ligands have been reported to promote lung inflammation and fibrosis, and to play a detrimental role in pathogen-induced pneumonia [3,7]. Recently, RAGE was reported to be involved in severe acute respiratory syndrome coronavirus 2 (SARS-CoV-2) infection [8]. This hypothesis developed from a study suggesting that angiotensin II (ATII) receptor type 1 (AT1R) can activate RAGE regardless of RAGE ligands [9]. SARS-CoV-2 uses the angiotensin-converting enzyme 2 (ACE2) as a receptor for cell invasion and, by inducing its downregulation, it increases ATII levels and AT1R activation [10,11]. Exosomes have been also shown to transfer ACE2 to recipient cells, thus contributing to spread of infection [12]. Recently, it was observed that plasma-derived extracellular vesicles from severe coronavirus disease-19 (COVID-19) patients are enriched in a newly identified RAGE binding protein (EN-RAGE) [13]. Furthermore, the gene encoding for this protein (S100A12) was upregulated in peripheral blood mononuclear cells from COVID-19 patients and strongly related to EN-RAGE protein levels in plasma [14].

Up to now, no studies have explored plasma levels of sRAGE and its forms in COVID-19 patients. Since the risk of severe hypoxia and death increases with increasing comorbidities like DM, in this study we evaluated how SARS-CoV-2 infection may affect these molecules in patients with and without DM and their potential role as biomarkers.

## 2. Experimental Section

### 2.1. Study Design and Participants

This is a retrospective observational study that involved 33 patients affected by COVID-19 admitted to the IRCCS Policlinico San Donato between 2 April and 25 April, 2020 and 99 healthy individuals (CTR). The diagnosis of SARS-CoV-2 infection was confirmed by real-time PCR on a nasopharyngeal swab specimen. A serum sample was taken at admission and stored at −80 °C. Patients were classified as affected by type 2 DM if they had a history of disease before enrolment which was previously diagnosed according to the following cut-off values: fasting glucose ≥ 126 mg/dL, HbA1c ≥ 48 mmol/mol, oral glucose tolerance test (75 g glucose) ≥ 200 mg/dL after 2 h, random glucose ≥ 200 mg/dL. Demographic, clinical, and biochemical data were obtained at admission. Biochemical parameters were assayed using Cobas 6000 analyzer (Roche Diagnostics, Milan, Italy) as previously reported [15,16]. The work was approved by Local Ethics Committee (75/INT/2020) and carried out in accordance with the Declaration of Helsinki. Patients signed a written informed consent.

### 2.2. Quantification of sRAGE, esRAGE and cRAGE

A measure of total sRAGE was obtained by the human ELISA kit from R&D Systems (Human RAGE Duo Set ELISA DY1145, Minneapolis, MN, USA) thich utilizes a monoclonal antibody that can detect both the cleaved and the alternatively spliced variant of RAGE (cRAGE and esRAGE, respectively). esRAGE was quantified by the ELISA assay from B-Bridged International (K1009-1, Santa Clara, CA, USA). This kit uses a monoclonal antibody able to bind only to esRAGE. The values of the intra- and inter-assay coefficient of variation were 6.37% and 4.78–8.97%, respectively. cRAGE was obtained by subtracting esRAGE from total sRAGE. cRAGE to esRAGE ratio (cRAGE/esRAGE) was than calculated. The GloMax^®^-Multi Microplate Multimode Reader was used for photometric measurements (Promega, Milan, Italy).

### 2.3. Quantification of Glycated Albumin

Glycated albumin (GA, g/L) and albumin were measured by the enzymatic QuantILab^®^ Glycated Albumin assay (Instrumentation Laboratory, Milan, Italy) using the ILab650 system (Instrumentation Laboratory). The percentage of glycated albumin (GA%) was automatically calculated by the ILab analyzer as GA/albumin ratio and corrected by an arithmetic algorithm that aligns the results to the HPLC reference method [17,18,19]. The minimum detectable concentration of GA is 1.15 g/L. The maximum intra- and inter-assay coefficient of variation values were 2.1% and 1.3% for GA and 1.2% and 1.0% for GA%, respectively.

### 2.4. Statistical Analysis

Qualitative variables are summarized as numbers and percentages. Quantitative variables are expressed as mean with standard deviation (SD). The normality of data distribution was assessed by the Kolmogorov–Smirnoff test. Differences across groups were compared by one-way ANOVA followed by Bonferroni post hoc test. Comparison between two groups was performed by unpaired *t*-test and Fisher exact tests, as appropriate. A *p*-value < 0.05 was considered as statistically significant. Analyses were performed using GraphPad Prism 5.0 biochemical statistical package (GraphPad Software, San Diego, CA, USA).

## 3. Results

The main characteristics of coronavirus disease-19 (COVID-19) patients and healthy individuals (CTR) are described in Table 1. COVID-19 patients are classified in diabetes mellitus (DM+) and not diabetes mellitus (DM).

Among the 33 patients, 5 were classified as having severe, 13 moderate, and 15 mild COVID-19. There was no evidence of difference in the classification of severity between non-DM and DM groups, as well as in the co-existence of other comorbidities and the use of drugs, except for antidiabetic drugs, which was greater in DM group. DM patients were older and with a lower estimated glomerular filtration rate (eGFR) than non-DM patients and CTR.

**Figure 1:**
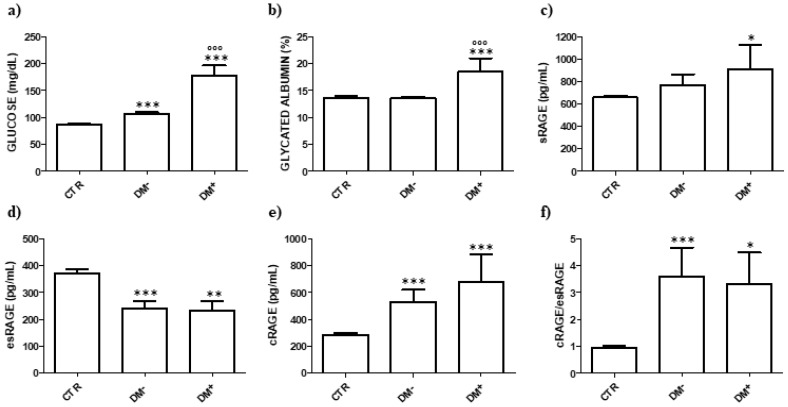
(**a**) Blood glucose, (**b**) glycated albumin, (**c**) sRAGE (soluble receptor for advanced glycation end products), (**d**) esRAGE (endogenously secretory receptor for advanced glycation end products), (**e**) cRAGE (membrane-cleaved receptor for advanced glycation end products), and (**f**) cRAGE/esRAGE ratio in COVID-19 (coronavirus disease-19) patients with and without diabetes mellitus (DM). CTR, healthy individuals; DM−, COVID-19 patients without DM; DM+, COVID-19 patients with DM. * *p* < 0.05 vs. CTR, ** *p* < 0.01 vs. CTR, *** *p* < 0.001 vs. CTR, °°° *p* < 0.001 vs. DM-.

Glucose levels were higher in both non-DM and DM COVID-19 patients compared with CTR (*p* < 0.001) and in DM compared with non-DM patients (*p* < 0.001) (Figure 1a). However, in non-DM group, glucose was higher than the cut-off point for DM diagnosis of 126 mg/dL just in three patients. GA was higher in DM patients compared with both CTR and non-DM patients (*p* < 0.001). There was no evidence of difference in the mean GA values between non-DM patients and CTR (Figure 1b).

We observed no evidence of difference between non-DM and DM groups in sRAGE, esRAGE and cRAGE levels, and cRAGE/esRAGE ratio (Figure 1c, 1d, 1e, and 1f, respectively). Mean sRAGE levels were higher in both non-DM and DM patients than in CTR, but the statistical significance was reached just in DM group (*p* < 0.05) (Figure 1c). esRAGE concentrations were lower in both non-DM (*p* < 0.001) and DM (*p* < 0.01) groups, while cRAGE levels were higher in both groups compared with CTR (*p* < 0.001) (Figure 1e). cRAGE/esRAGE ratio had the same trend of cRAGE (*p* < 0.001 for non-DM vs. CTR and *p* < 0.05 for DM vs. CTR) (Figure 1f).

## 4. Discussion

DM patients affected by COVID-19 are at high risk of acute respiratory distress, severe hypoxia, and death [20]. In DM patients, chronically activated RAGE axis was suggested as one of the potential mechanisms that increase the risk of developing severe COVID-19 and having a worse outcome [8,20]. In patients with DM, the high levels of AGE and other RAGE ligands can promote pro-inflammatory and a pro-thrombotic states, endothelial cells dysfunction, and vascular leakage, thus predisposing to further damage by SARS-CoV-2 infection [8,21,22].

It is known that any noxa affecting RAGE can also modulate the soluble forms of the receptor. However, how SARS-CoV-2 infection, alone or in association with DM, may affect them has never been explored before now.

We observed, for the first time, that in COVID-19 patients, sRAGE, esRAGE, cRAGE, and cRAGE/esRAGE ratio show a trend that seems to be independent of DM. DM patients had higher fasting plasma glucose and GA levels, which confirm the diagnosis of DM. Although non-DM patients have higher glucose levels than CTR, this increase was within the physiological range, except for three patients. GA, which is a medium-term retrospective marker of glucose metabolism (about 20 days), confirmed that patients classified as non-DM did not experience hyperglycemia prior to admission to first aid.

sRAGE levels and its forms are known to be increased in patients with DM [4]. sRAGE is a circulating pool composed of cRAGE, which derives from the proteolytic cleavage of the membrane-bound RAGE, and esRAGE, a splice variant which contains just the extracellular domains [23]. Activation of membrane RAGE promotes inflammation and upregulates inflammatory enzymes, such as metalloproteases, that, by cleaving RAGE, increase cRAGE levels [24]. The reduced availability of membrane RAGE and the increased levels of circulating cRAGE are considered two important counter-regulatory mechanisms to protect against RAGE activation [25,26]. In addition to sRAGE, DM patients have also higher serum levels of ADAM 10 (A disintegrin and metalloproteinase 10), a metalloprotease which was found to correlate with serum levels of cRAGE [27]. Considering that the activation of a pro-inflammatory response and of counter-regulatory mechanisms that protect against RAGE ligands are the main mechanisms affecting the levels of sRAGE and its forms, we can suppose that the same pathways may be activated also during SARS-CoV-2 infection. Notably, increased levels of specific RAGE ligands have been observed in COVID-19 patients [13,14]. However, we cannot exclude that the increased levels of cRAGE depend on the activation of other mechanisms modulated by the virus during cell invasion, such as ACE2 downregulation. Considering sRAGE and its forms, we expected to observe this scenario in DM patients because it reflects the activation and modulation of the RAGE system due to hyperglycemia and increased AGE levels. Maybe, the higher age and the worse kidney function observed in DM patients are additional factors that can further increase sRAGE and cRAGE, and downregulate esRAGE levels in this group. Nevertheless, it is worth noting that the levels of sRAGE, esRAGE, cRAGE and cRAGE/esRAGE ratio did not differ between DM and non-DM patients, despite the co-existence of additional factors that can affect the RAGE axis in DM group. The only slight difference between non-DM and DM patients was observed by comparing sRAGE levels to those of CTR. In fact, in non-DM patients the rise of sRAGE levels did not reach the statistical significance. However, the observation that the specific forms increase or decrease significantly in both groups compared with CTR confirms the existence of the same trend. Therefore, SARS-CoV-2 infection seems to play a major role in modulating the circulating levels of these molecules regardless of glycemic control and other factors that can additionally affect the same pathway.

Up to now, the few data existing on RAGE and COVID-19 have just focused on the potential pathological role of RAGE axis in lung inflammation, disease onset, and progression [8,14,21,28]. We did not explore the potential molecular mechanisms linking SARS-CoV-2 infection and changes in the levels of sRAGE and its forms. Maybe, as previously supposed, changes in ATII levels during infection could activate the RAGE pathway and amplify the pro-inflammatory response [9], but this needs further investigation. Since we just focused on the soluble forms of the receptors and we did not explore membrane RAGE and its ligand, we can not make any conclusion that confirms or refuses the pivotal role of RAGE in worsening the clinical outcome of patients affected by DM, as previously supposed.

Our study is of novelty because it opens to the possibility to look at sRAGE and its forms as potential disease biomarkers for COVID-19. We know that the circulating levels of these molecules may be affected by different diseases [29], but the observation that the overall trend was the same regardless of the existence of comorbidities (i.e., DM) and other confounding factors (i.e., age and kidney function) emphasizes the potential of these molecules in the clinical management of COVID-19.

In our opinion, cRAGE/esRAGE ratio could be of particular interest as a biomarker because it integrates the levels of the two different forms [24,30]. Being the two molecules produced by different mechanisms [24,31], the ratio can be better than the single forms because it suggests how they can be proportionally or disproportionally affected at the same time.

Our study has the limitation of being an observational study that includes a low number of patients. Moreover, the small number of severe patients did not allow us to consider any potential link between disease’s severity and the levels of sRAGE and its forms. No conclusion about the potential use of these molecules as biomarkers can be drawn now, but the results encourage longitudinal studies to further explore their role.

## 5. Conclusions

In conclusion, our data seem to support the hypothesis that COVID-19 can activate and modulate the RAGE system regardless of other factors. Identifying the role of sRAGE and its forms as biomarkers, their association to COVID-19 prognosis, and also the potential of the RAGE pathway as a therapeutic target seem of relevance to control inflammatory burden and to help treatment of COVID-19.

## Figures and Tables

**Table 1:** Patient’s characteristics.

	DM+	DM−	CTR
*N*	11	22	99
Age, years	72.6 (15.8) *^,^°	55.6 (22.5) °	43.5 (11.5)
Males (*n*, %)	8 (72.7)	13 (59)	53 (53.5)
COVID-19 Severity			
Mild (*n*, %)	4 (36.4)	11 (50.0)	-
Moderate (*n*, %)	6 (54.5)	7 (31.8)	-
Severe (*n*, %)	1 (9.1)	4 (18.2)	-
Other comorbidities			
Hypertension (*n*, %)	5 (45.5)	6 (27.3)	-
History of cardiovascular disease (*n*, %)	4 (36.4)	2 (9.1)	-
Chronic renal disease (*n*, %)	1 (9.1)	1 (4.5)	-
COPD (*n*, %)	1 (9.1)	1 (4.5)	-
Smoking (*n*, %)	1 (9.1)	-	-
Biochemical data			
CRP (mg/dL)	5.5 (5.6)	5.8 (6.6)	-
eGFR (mL/min/1.73 m^2^)	58.4 (20.6) *^,^°	73.6 (23.4)	84.0 (8.9)
White blood cell count (×1000/µL)	8.90	7.20	-
Drugs			
Antidiabetic drugs (*n*, %)	11 (100) ^+,^°	-	-
Aspirin (*n*, %)	3 (27.3)	2 (9.1)	-
ACEI/ARB (*n*, %)	2 (18.2)	4 (18.2)	-
β-Blockers (*n*, %)	3 (27.3)	4 (18.2)	-
Calcium channel blokers (*n*, %)	1 (9.1)	3 (13.6)	-
Statins (*n*, %)	3 (27.3)	1 (4.5)	-

Data are expressed as number or percentage or mean and (SD). ACEI: angiotensinogen-converting enzyme inhibitor; ARB: angiotensin receptor blockade; CRP, C reactive protein; COPD, chronic obstructive pulmonary disease; eGFR, estimated glomerular filtration rate; N, number. * *p* < 0.05, ^+^ *p* < 0.001 vs. DM−; ° *p* < 0.001 vs. controls (CTR).

## References

[1] Schmidt A.M. (2015). Soluble RAGEs—Prospects for treating & tracking metabolic and inflammatory disease. Vasc. Pharm..

[2] Egana-Gorrono L., Lopez-Diez R., Yepuri G., Ramirez L.S., Reverdatto S., Gugger P.F., Shekhtman A., Ramasamy R., Schmidt A.M. (2020). Receptor for Advanced Glycation End Products (RAGE) and Mechanisms and Therapeutic Opportunities in Diabetes and Cardiovascular Disease: Insights From Human Subjects and Animal Models. Front. Cardiovasc. Med..

[3] Oczypok E.A., Perkins T.N., Oury T.D. (2017). All the “RAGE” in lung disease: The receptor for advanced glycation endproducts (RAGE) is a major mediator of pulmonary inflammatory responses. Paediatr. Respir. Rev..

[4] Selvin E., Halushka M.K., Rawlings A.M., Hoogeveen R.C., Ballantyne C.M., Coresh J., Astor B.C. (2013). sRAGE and risk of diabetes, cardiovascular disease, and death. Diabetes.

[5] Dozio E., Vianello E., Briganti S., Lamont J., Tacchini L., Schmitz G., Corsi Romanelli M.M. (2016). Expression of the Receptor for Advanced Glycation End Products in Epicardial Fat: Link with Tissue Thickness and Local Insulin Resistance in Coronary Artery Disease. J. Diabetes Res..

[6] Dozio E., Vianello E., Sitzia C., Ambrogi F., Benedini S., Gorini S., Rampoldi B., Rigolini R., Tacchini L., Romanelli M.M.C. (2020). Circulating Irisin and esRAGE as Early Biomarkers of Decline of Metabolic Health. J. Clin. Med..

[7] Boteanu R.M., Uyy E., Suica V.I., Antohe F. (2015). High-mobility group box 1 enhances the inflammatory process in diabetic lung. Arch. Biochem. Biophys..

[8] Rojas A., Gonzalez I., Morales M.A. (2020). SARS-CoV-2-mediated inflammatory response in lungs: Should we look at RAGE?. Inflamm. Res..

[9] Pickering R.J., Tikellis C., Rosado C.J., Tsorotes D., Dimitropoulos A., Smith M., Huet O., Seeber R.M., Abhayawardana R., Johnstone E.K. (2019). Transactivation of RAGE mediates angiotensin-induced inflammation and atherogenesis. J. Clin. Investig..

[10] Zhang S., Liu Y., Wang X., Yang L., Li H., Wang Y., Liu M., Zhao X., Xie Y., Yang Y. (2020). SARS-CoV-2 binds platelet ACE2 to enhance thrombosis in COVID-19. J. Hematol. Oncol..

[11] Zhao P., Praissman J.L., Grant O.C., Cai Y., Xiao T., Rosenbalm K.E., Aoki K., Kellman B.P., Bridger R., Barouch D.H. (2020). Virus-Receptor Interactions of Glycosylated SARS-CoV-2 Spike and Human ACE2 Receptor. Cell Host Microbe.

[12] Wang J., Chen S., Bihl J. (2020). Exosome-Mediated Transfer of ACE2 (Angiotensin-Converting Enzyme 2) from Endothelial Progenitor Cells Promotes Survival and Function of Endothelial Cell. Oxid. Med. Cell. Longev..

[13] Krishnamachary B., Cook C., Spikes L., Chalise P., Dhillon N.K. (2020). The Potential Role of Extracellular Vesicles in COVID-19 Associated Endothelial injury and Pro-inflammation. medRxiv.

[14] Arunachalam P.S., Wimmers F., Mok C.K.P., Perera R., Scott M., Hagan T., Sigal N., Feng Y., Bristow L., Tak-Yin Tsang O. (2020). Systems biological assessment of immunity to mild versus severe COVID-19 infection in humans. Science.

[15] Malavazos A.E., Corsi M.M., Ermetici F., Coman C., Sardanelli F., Rossi A., Morricone L., Ambrosi B. (2007). Proinflammatory cytokines and cardiac abnormalities in uncomplicated obesity: Relationship with abdominal fat deposition. Nutr. Metab. Cardiovasc. Dis..

[16] Benedini S., Dozio E., Invernizzi P.L., Vianello E., Banfi G., Terruzzi I., Luzi L., Corsi Romanelli M.M. (2017). Irisin: A Potential Link between Physical Exercise and Metabolism-An Observational Study in Differently Trained Subjects, from Elite Athletes to Sedentary People. J. Diabetes Res..

[17] Kouzuma T., Usami T., Yamakoshi M., Takahashi M., Imamura S. (2002). An enzymatic method for the measurement of glycated albumin in biological samples. Clin. Chim. Acta.

[18] Kouzuma T., Uemastu Y., Usami T., Imamura S. (2004). Study of glycated amino acid elimination reaction for an improved enzymatic glycated albumin measurement method. Clin. Chim. Acta.

[19] Kohzuma T., Yamamoto T., Uematsu Y., Shihabi Z.K., Freedman B.I. (2011). Basic performance of an enzymatic method for glycated albumin and reference range determination. J. Diabetes Sci. Technol..

[20] Roncon L., Zuin M., Rigatelli G., Zuliani G. (2020). Diabetic patients with COVID-19 infection are at higher risk of ICU admission and poor short-term outcome. J. Clin. Virol..

[21] De Francesco E.M., Vella V., Belfiore A. (2020). COVID-19 and Diabetes: The Importance of Controlling RAGE. Front. Endocrinol..

[22] Varga Z., Flammer A.J., Steiger P., Haberecker M., Andermatt R., Zinkernagel A.S., Mehra M.R., Schuepbach R.A., Ruschitzka F., Moch H. (2020). Endothelial cell infection and endotheliitis in COVID-19. Lancet.

[23] Schlueter C., Hauke S., Flohr A.M., Rogalla P., Bullerdiek J. (2003). Tissue-specific expression patterns of the RAGE receptor and its soluble forms--a result of regulated alternative splicing?. Biochim. Biophys. Acta.

[24] Raucci A., Cugusi S., Antonelli A., Barabino S.M., Monti L., Bierhaus A., Reiss K., Saftig P., Bianchi M.E. (2008). A soluble form of the receptor for advanced glycation endproducts (RAGE) is produced by proteolytic cleavage of the membrane-bound form by the sheddase a disintegrin and metalloprotease 10 (ADAM10). FASEB J..

[25] Zhang L., Bukulin M., Kojro E., Roth A., Metz V.V., Fahrenholz F., Nawroth P.P., Bierhaus A., Postina R. (2008). Receptor for advanced glycation end products is subjected to protein ectodomain shedding by metalloproteinases. J. Biol. Chem..

[26] Selejan S.R., Hewera L., Hohl M., Kazakov A., Ewen S., Kindermann I., Bohm M., Link A. (2017). Suppressed MMP-9 Activity in Myocardial Infarction-Related Cardiogenic Shock Implies Diminished Rage Degradation. Shock.

[27] Lee A.C., Lam J.K., Shiu S.W., Wong Y., Betteridge D.J., Tan K.C. (2015). Serum Level of Soluble Receptor for Advanced Glycation End Products Is Associated with A Disintegrin And Metalloproteinase 10 in Type 1 Diabetes. PLoS ONE.

[28] Stilhano R.S., Costa A.J., Nishino M.S., Shams S., Bartolomeo C.S., Breithaupt-Faloppa A.C., Silva E.A., Ramirez A.L., Prado C.M., Ureshino R.P. (2020). SARS-CoV-2 and the possible connection to ERs, ACE2, and RAGE: Focus on susceptibility factors. FASEB J..

[29] Vazzana N., Santilli F., Cuccurullo C., Davi G. (2009). Soluble forms of RAGE in internal medicine. Intern. Emerg. Med..

[30] Hudson B.I., Moon Y.P., Kalea A.Z., Khatri M., Marquez C., Schmidt A.M., Paik M.C., Yoshita M., Sacco R.L., DeCarli C. (2011). Association of serum soluble receptor for advanced glycation end-products with subclinical cerebrovascular disease: The Northern Manhattan Study (NOMAS). Atherosclerosis.

[31] Hudson B.I., Carter A.M., Harja E., Kalea A.Z., Arriero M., Yang H., Grant P.J., Schmidt A.M. (2008). Identification, classification, and expression of RAGE gene splice variants. FASEB J..

